# Sikri’s Magnetic Attachment Retained Two-Tray (SMART) impression technique for prosthodontic management of a knife-edge residual ridge: a case report

**DOI:** 10.2340/biid.v13.46765

**Published:** 2026-09-11

**Authors:** Arpit Sikri, Jyotsana Sikri, Rekha Thiruvengadam, Muthu Thiruvengadam

**Affiliations:** a Department of Prosthodontics and Crown & Bridge, Punjab Government Dental College and Hospital, Amritsar, Punjab, India; b Department of Conservative Dentistry & Endodontics, Bhojia Dental College & Hospital, Budh (Baddi), Teh. Baddi, Distt. Solan, Himachal Pradesh, India; c Microbiology and Infectious Diseases - Helix Research Lab, Department of Neonatology, Saveetha Medical College and Hospital, Saveetha Institute of Medical and Technical Sciences (SIMATS), Chennai, India, Chennai, 602105, India; d Department of Applied Bioscience, College of Life and Environmental Science, Konkuk University, Seoul, Republic of Korea

**Keywords:** Dual tray, knife-edge ridge, magnetically retained custom tray, modified custom tray, mucostatic impression, sharp residual ridge, Sikri’s technique, SMART tray impression technique, split tray, thin ridge

## Abstract

**Background:**

Mandibular knife-edge residual ridges present a significant challenge during complete denture impression making because of their narrow morphology and susceptibility to tissue distortion under pressure. This report describes the clinical application of the novel Sikri’s Magnetic Attachment Retained Two-Tray (SMART) impression technique, conceptualized, developed, and patented by Dr. Arpit Sikri (Patent/ROC No. L-156844/2024), for obtaining an accurate mucostatic impression of a mandibular knife-edge residual ridge without the application of manual finger pressure. The SMART technique employs a magnetically retained split custom tray design that facilitates pressure-free tray stabilization and precise impression making in anatomically challenging mandibular residual ridges.

**Materials and Methods:**

A completely edentulous patient with a pronounced knife-edge residual ridge in the anterior mandible was rehabilitated using the SMART impression technique. A custom-designed split two-part impression tray incorporating magnetic attachments for self-retention and retentive holes for impression material retention was fabricated. Magnetic attraction between the tray components ensured stable and reproducible tray positioning throughout the impression procedure, eliminating the need for operator-applied finger pressure and facilitating a pressure-free mucostatic impression of the residual ridge.

**Results:**

The SMART technique enabled accurate recording of the mandibular residual ridge anatomy without observable tissue displacement during impression making. The definitive impression produced a well-adapted master cast, resulting in a complete denture with satisfactory retention, stability, adaptation, and patient comfort during clinical evaluation.

**Conclusion:**

The SMART impression technique represents a simple, innovative, and clinically applicable approach for the prosthodontic management of mandibular knife-edge residual ridge. By eliminating manual tray stabilization and minimizing tissue displacement during impression making, it facilitates accurate mucostatic impressions and may improve denture fit, stability, and overall clinical performance. Further clinical studies involving larger patient populations are required to evaluate its reproducibility and long-term clinical effectiveness.

## Graphical abstract

**Figure:**
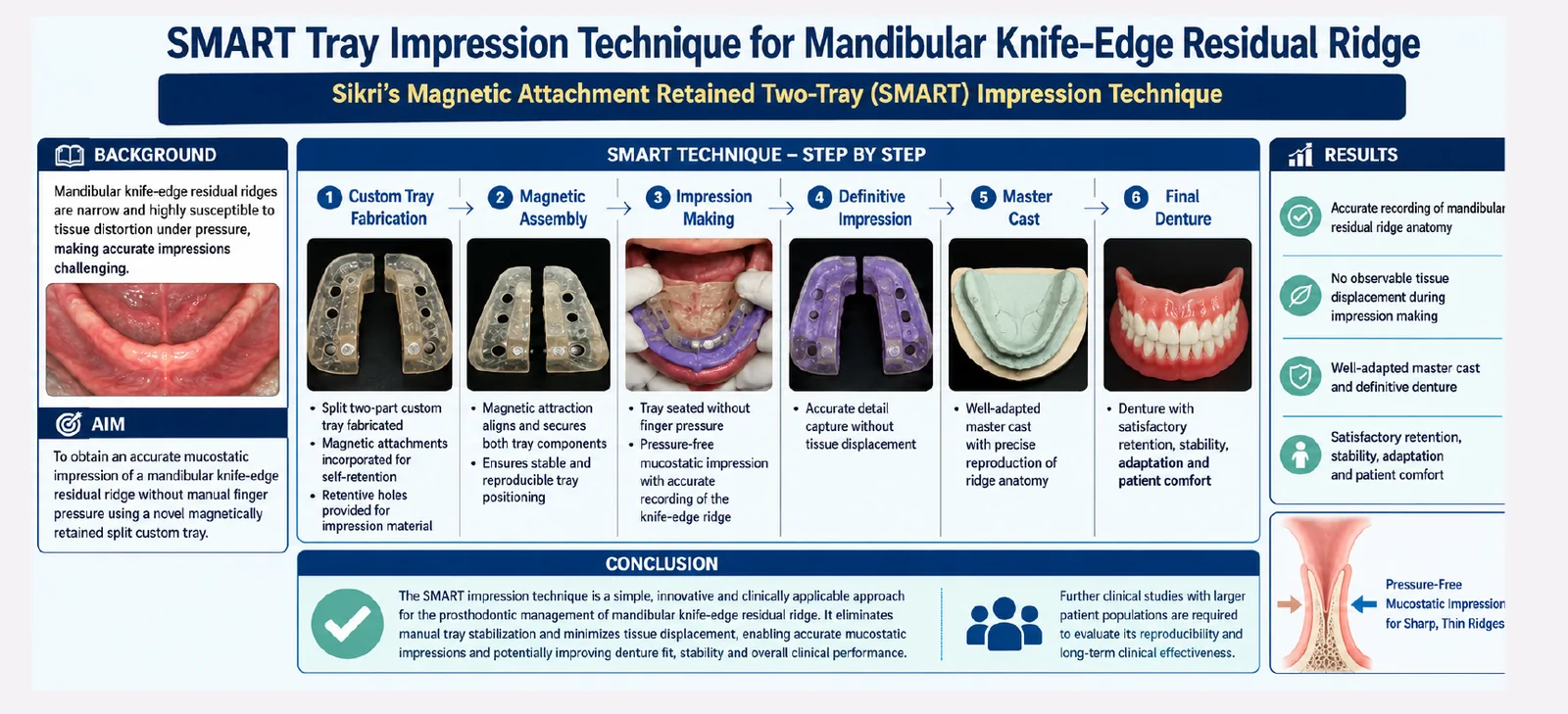
GRAPHICAL ABSTRACT

**KEY MESSAGES**

1. SMART magnetic split-tray impressions accurately record knife-edge ridges while minimizing tissue displacement.
2. Magnetic self-alignment of the split custom tray eliminates the need for operator finger pressure, enabling controlled tray positioning and more accurate mucostatic impressions.
3. Pressure-free mucostatic impressions improve denture stability, retention, comfort, and clinical outcomes.

## Introduction

Tooth loss is a natural progression that is often associated with dental caries and periodontal diseases. After a tooth is lost, the alveolar bone referred to as the residual alveolar ridge undergoes changes, a process known as residual ridge resorption (RRR) [1]. RRR is a chronic, progressive, irreversible, and complex biophysical process that affects nearly all patients [2]. The term describes the reduction in both the quantity and quality of the residual ridge following tooth extraction (as defined by GPT 10) [3]. Comparatively, the edentulous mandible experiences a greater degree of RRR than the edentulous maxilla, with a mean ratio of 1:4.2 between maxillary anterior and mandibular anterior RRR [4]. As a result, the ridge morphology flattens due to accelerated resorption along its crest. In some cases, such as those with knife-edge ridges, lateral resorption causes the ridge to shrink buccolingually rather than vertically. Additionally, the residual alveolar ridge may lose its density and be replaced by mobile soft tissue, commonly referred to as flabby tissue [5].

A knife-edge ridge is a type of residual alveolar ridge characterized by significant buccolingual resorption with rapid lateral degradation of the buccal and lingual regions of the ridge. This condition predominantly affects the mandibular edentulous ridge, particularly in the anterior region, with an occurrence rate of 89% [6]. It is commonly observed in edentulous patients. Other terms used to describe this condition include saw-tooth ridge, razor-thin ridge, sharp residual ridge, thin, wiry, or spiny ridge.

Patients with a knife-edge ridge often report chronic and persistent pain beneath their dentures, which is typically localized to areas of irritation. They may experience intense discomfort, particularly during activities, such as mastication or tooth clenching. Chronic irritation and soreness over the underlying edentulous residual ridges are common complaints, and the sharp contours of the ridges can exacerbate the discomfort caused by dentures. Goodsell highlighted that one of the primary reasons patients struggle with their dentures is the presence of razor-like or saw-tooth-shaped ridges [7]. Similarly, Landa observed that denture failures are notably more frequent when the mandibular ridge has a short, pointed configuration [8].

The formation of a knife-edge ridge can be attributed to multiple factors, with each patient exhibiting a unique degree of bone resorption. When several contributing factors converge, significant bone resorption may occur. Atwood’s classification system, which consists of six orders, categorizes the configuration of the residual alveolar ridge and places the knife-edge ridge under Order IV [1]. This classification is characterized by a marked narrowing of the labio-lingual diameter at the ridge crest, accompanied by compensatory internal remodeling that further sharpens the ridge. Such sharp ridges often develop because of immediate denture placement. Furthermore, research suggests that postmenopausal women are more susceptible to developing a knife-edge ridge in the mandibular region [9].

Accurate diagnosis of a knife-edge residual ridge requires comprehensive evaluation and meticulous treatment planning, which includes reviewing the patient’s medical and dental history, conducting a thorough clinical examination, and performing precise radiographic interpretation. Radiographic analysis is the most effective method to detect sharp bony projections on the crest of the residual alveolar ridge. Three principal forms of sharp residual ridges have been identified radiographically: sawtooth ridges, razor-like ridges, and ridges with prominent spiny projections [6]. Each of these ridge morphologies may cause pain and discomfort beneath complete dentures, thereby compromising denture retention, stability, and patient comfort.

Several therapeutic approaches are available for treating knife-edge ridges, encompassing both surgical and non-surgical methods. Surgical correction aims to create a smooth, pain-free, denture-bearing surface that can withstand normal masticatory forces. As Bear aptly noted, ‘The success of any functional and aesthetically pleasing restoration is inherently linked to its foundational support’ [10]. For some patients, an interim solution may involve adjusting or relieving the denture in areas directly over the sharp ridges. Bolender and Swenson reported positive outcomes with vestibular extension procedures [11], while Thoma suggested using tantalum gauze or gelatin sponge on the labial side of sharp ridges particularly in cases involving undercuts to encourage new tissue formation and avoid bone removal [12]. Silicone rubber has also been employed in ridge extension techniques [13]. While ridge augmentation with bone grafts can be an adjunct during surgery or implant placement, its effectiveness remains debated.

The choice of treatment depends on several factors, including patient willingness, age, medical condition, procedural duration, potential discomfort, risk of surgical complications, implant failure rates, treatment predictability, and associated costs. Consequently, there is a growing preference for nonsurgical and prosthetic rehabilitation methods. As DeVan famously stated, ‘Perpetual preservation of what remains is more important than the meticulous replacement of what is lost’ [14]. The prosthodontic or non-surgical approach relies on conventional prosthodontic techniques, thus avoiding the need for surgery. This method involves the selection of appropriate impression materials and techniques to fabricate a complete denture that accommodates the knife-edge ridge. This case report introduces the novel Sikri’s Magnetic Attachment Retained Two-Tray (SMART) impression technique for the prosthodontic management of mandibular knife-edge residual ridges. The technique employs a custom-designed split two-part impression tray incorporating magnetic attachments for self-retention and retentive holes for impression material retention. Magnetic self-retention stabilizes the tray without the need for operator-applied finger pressure during impression making, thereby facilitating a pressure-free mucostatic impression and minimizing tissue displacement.

The aim of this case report is to describe the clinical application of the SMART impression technique in the management of a mandibular knife-edge residual ridge. By accurately recording the delicate residual ridge anatomy under mucostatic conditions, the technique may enhance denture support, stability, retention, adaptation, and patient comfort, while providing a simple, reproducible, and clinically applicable approach for managing this challenging clinical condition.

## Case presentation

A 62-year-old female patient presented with complaints of difficulty eating due to missing teeth for the past 2 years. Her medical history was unremarkable and she had never worn any removable dental prostheses. Radiographic evaluation revealed significant bone resorption leading to a marked reduction in both the height and width of the mandibular bone. Intraoral examination revealed a complete absence of teeth in both the maxillary and mandibular arches, along with a pronounced knife-edge ridge in the mandibular arch and extensive erythema of the mucosal membrane. The patient was informed of various treatment options, including conventional prosthodontic rehabilitation, surgical interventions, and implant-supported fixed or removable prostheses. Due to financial limitations, the decision was made to utilize a novel impression technique, specifically the SMART impression technique, to fabricate a conventional complete denture. Written informed consent was obtained from the patient for publication of this case report and the accompanying clinical photographs.

This case report aims to introduce an innovative technique for creating a complete mucostatic impression of a mandibular knife-edge ridge using magnets. This method offers a simple and effective approach for managing the complex knife-edge ridge in the mandibular arch. It involves the use of a split two-part custom tray incorporating magnetic attachments for self-retention and retentive holes for impression material retention. The magnetic self-retention eliminates the need for operator-applied finger pressure during impression making, thereby facilitating a pressure-free mucostatic impression. The primary goal was to address the challenges posed by knife-edge ridges in the mandibular arch. The patient provided informed written consent for treatment using this novel impression technique and for the potential publication of the case in future studies.

## Clinical procedure

**Figure 1:**
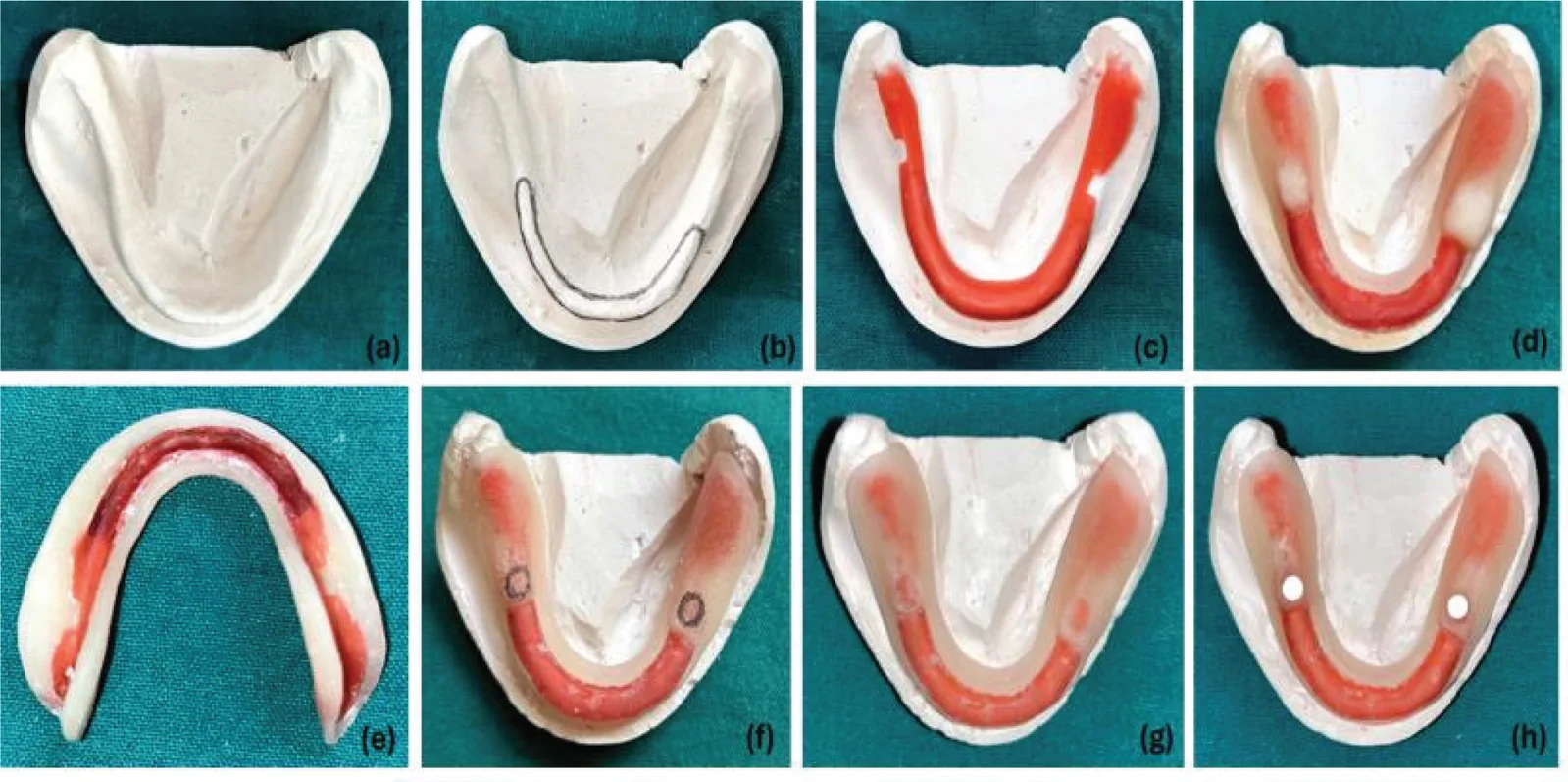
Fabrication of the primary cast and custom tray preparation (a–h). (a) Fabrication of the primary cast. (b) Demarcation of the knife-edge ridge region on the primary cast. (c) Adaptation of a double-thickness wax spacer over the outlined area. (d) Occlusal view showing fabrication of Tray-1. (e) Intaglio surface of Tray-1 illustrating spacer adaptation. (f) Proposed markings for magnetic disc positioning. (g) Formation of recesses on Tray-1 to accommodate magnetic discs. (h) Placement of ferrite magnetic discs within the prepared depressions on Tray-1.

The initial stages of complete denture fabrication followed standard procedures, including examination, diagnosis, preliminary and final impressions, recording maxillo-mandibular relations, tooth selection, wax trial denture try-in, and delivery of the final prosthesis. Preliminary impressions of both the maxillary and mandibular arches were obtained using a medium-fusing impression compound (Hiflex Impression Compound; Prevest DenPro Limited, Jammu, India). These impressions were carefully beaded and boxed using baseplate wax (MAARC Dental, Maharashtra, India) and then poured with type II dental plaster (GypRock plaster, Rajkot, Gujarat, India) to produce an accurate primary cast (Figure 1a). The knife-edge ridge was clearly delineated on the preliminary cast (Figure 1b). To ensure proper relief over the knife-edge ridge area, a double-thickness wax spacer was meticulously applied (Figure 1c).

### Fabrication of SMART Tray

A custom tray with magnetic retention was designed to achieve complete final mucostatic impression of the mandibular arch. The custom tray was fabricated in two parts: Tray-1 (first part) and Tray-2 (second part). The trays were divided into two sections to address the different regions of the arch. Region-1, which was relatively stable, included anatomical landmarks excluding the knife-edge ridge area, while Region-2 specifically covered the knife-edge ridge. Tray-1 was used for the non-knife-edge ridge regions and Tray-2 was dedicated to the knife-edge ridge area, ensuring precise and controlled impression making for both regions.

### Tray-1 (Non-knife-edge ridge) fabrication

A single-thickness layer of modeling wax (MAARC Dental, Maharashtra, India) was applied in region-1. Tray-1 was then fabricated using an auto-polymerizing acrylic resin (DPI RR Cold Cure, Dental Products of India, Mumbai, India), ensuring that the tray was 2 mm below the sulcus depth. In this design, the wax layer was positioned 4 mm short of the sulcus, with the final tray extending to 2 mm short of the sulcus (Figure 1d and e). Two depressions were created in Tray-1 to accommodate ferrite magnetic discs (6-mm diameter) (M/S Sidhi Enterprises, New Delhi, India) with a magnetic field strength of 0.35 tesla, which were placed and securely embedded within the tray (Figure 1f–h).

### Tray-2 (Knife-edge ridge) fabrication

**Figure 2:**
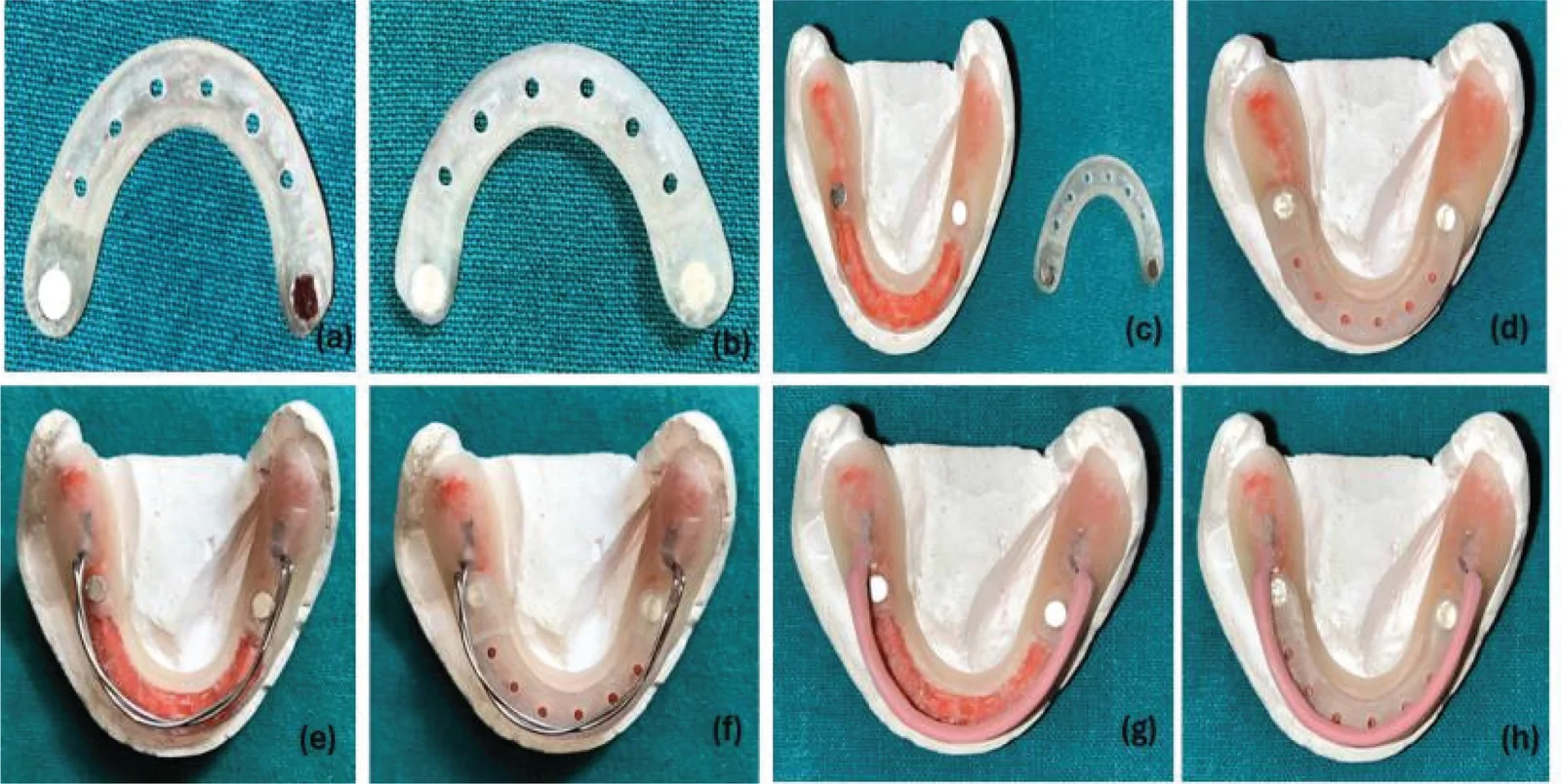
Fabrication and assembly of the SMART dual-tray system (a–h). (a) Intaglio surface of Tray-2 following fabrication. (b) Occlusal surface of the completed Tray-2. (c) Individual components of Tray-1 and Tray-2 prior to assembly. (d) Magnetic coupling between Tray-1 and Tray-2 demonstrating precise alignment. (e) Contouring of 19-gauge double-thickness wire on the primary cast. (f) Securing of retentive wire tags in their designated positions. (g) Beading of the 19-gauge wire with acrylic resin for reinforcement. (h) Final configuration of the ergonomically designed SMART tray handle.

Two layers of modeling wax (MAARC Dental, Maharashtra, India) were applied, and the tray was extended to ensure proper alignment of the two rare earth circular magnets with the magnets in Tray-1. Tray-2 was then fabricated using an autopolymerizing acrylic resin (DPI RR Cold Cure, Dental Products of India, Mumbai, India), covering the knife-edge ridge area in Region-2 (Figure 2a, b).

### Tray assembly

The magnets were secured in place using an autopolymerizing acrylic resin. Trays 1 and 2 (Figure 2c) were then positioned in their respective areas on the primary cast. When assembled, the resin extensions of Tray-2 engaged with the depressions in Tray-1, thereby ensuring alignment. The custom tray remained stable because of the magnetic attraction between the opposing poles of Tray-1 and Tray-2. The margins of both trays were beveled at their contact points to ensure a close fit during seating. Finally, Trays 1 and 2 were positioned on the primary cast in their designated areas (Figure 2d).

### SMART tray handle design

An innovative tray handle design, known as SMART tray handle design, was introduced. A double-thickness 19-gauge round hard temper wire (Smith Stainless Steel Wire, K.C. SMITH & Co., England) was contoured to match the shape of the residual ridge on the primary cast (Figure 2e). Both ends of the wire, known as retentive tags, were placed in their designated positions and secured using an auto-polymerizing acrylic resin (DPI RR Cold Cure, Dental Products of India, Mumbai, India) (Figure 2f). The wire was then beaded with an auto-polymerizing acrylic resin (Figure 2g) and finger rests were fabricated, typically around the first molar region. These rests or stops were strategically placed to stabilize the tray and prevent movement or sliding during the final impression. The design of the finger rests included a small, sharp portion to help assess the pressure applied during the impression-making procedure. This led to the creation of the SMART tray handle design (Figure 2h), which facilitated the easy handling of the tray in the patient’s mouth. It allowed the patient to perform unobstructed tongue and various functional movements during border molding and final impression procedures, aiding in effective tray manipulation.

### SMART tray impression technique

**Figure 3:**
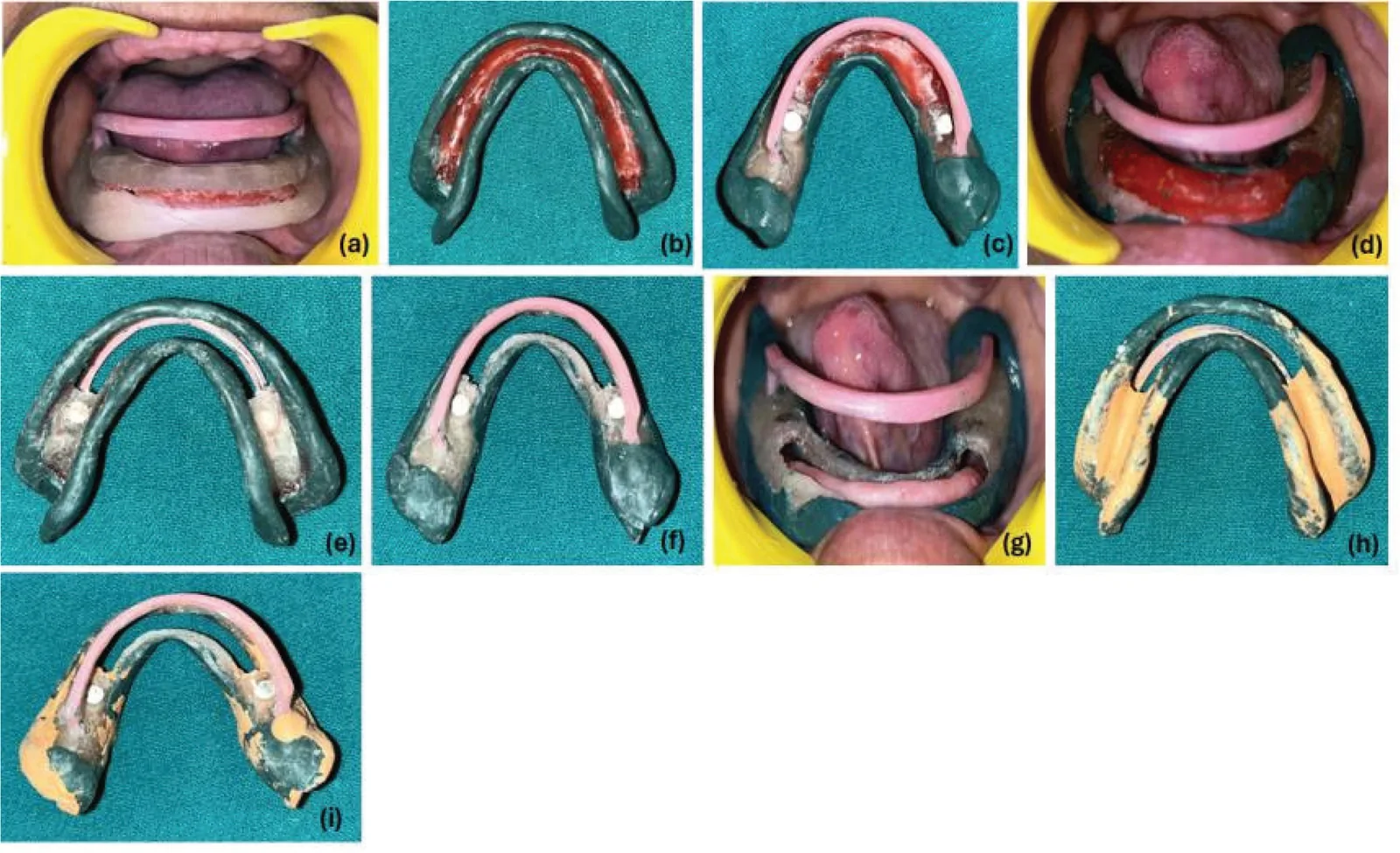
Clinical evaluation and border-molding procedure using the SMART tray (a–i). (a) Intraoral evaluation of the assembled SMART magnetic tray. (b) Intaglio view depicting border-molding on Tray-1. (c) Occlusal view of the border-molding process on Tray-1. (d) Border-molding procedure performed intraorally. (e) Intaglio view of Tray-1 following wax-spacer removal. (f) Occlusal view of Tray-1 after spacer removal. (g) Intraoral verification of Tray-1 adaptation following spacer removal. (h) Intaglio view of the definitive impression made with Tray-1. (i) Occlusal view of the final impression illustrating uniform detail reproduction.

**Figure 4:**
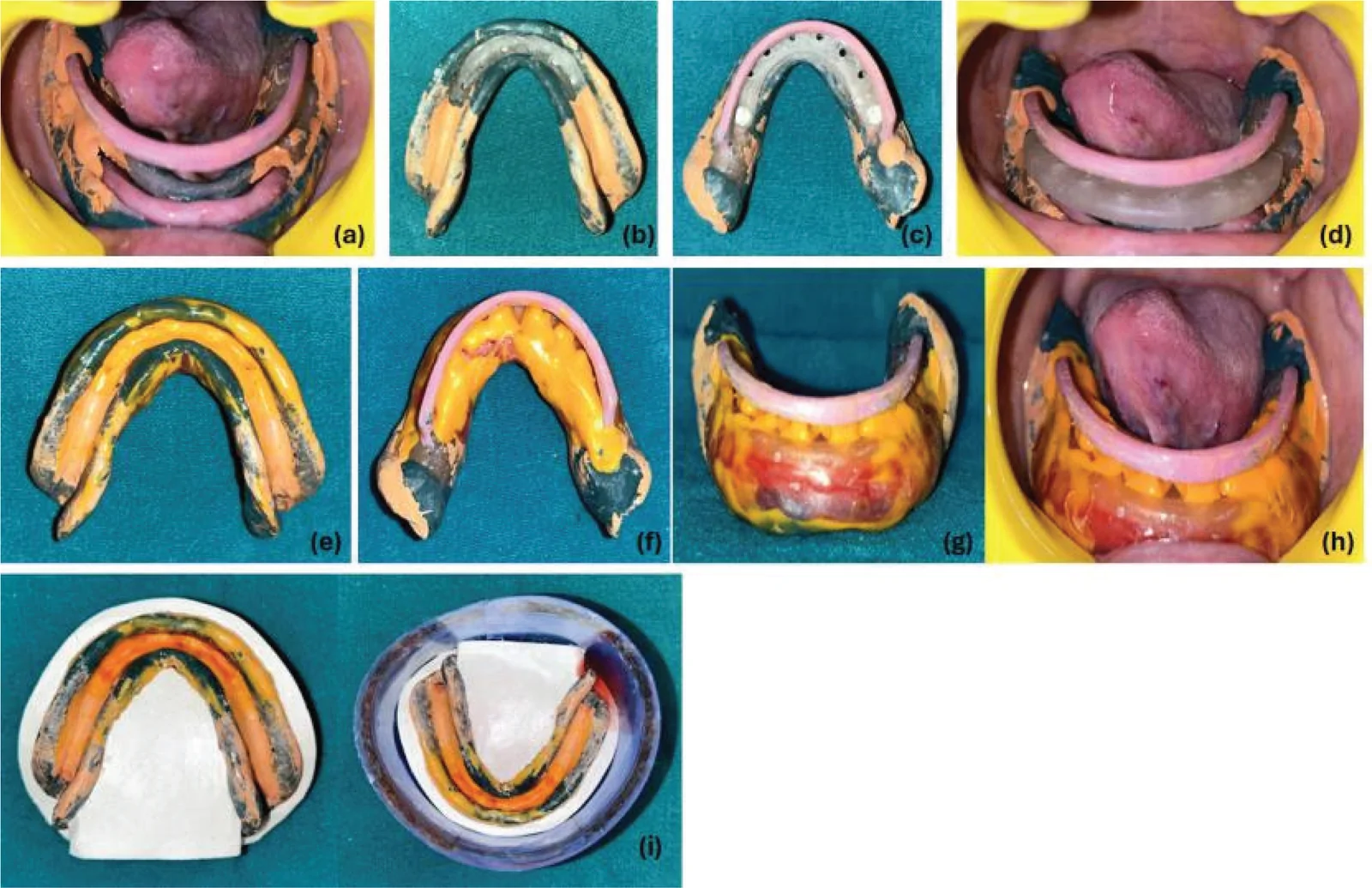
Final SMART tray impression and cast preparation (a–h). (a) Intraoral evaluation of the completed definitive impression. (b) Placement of Tray-2 on Tray-1 after impression making – intaglio view. (c) Placement of Tray-2 on Tray-1 after impression making – occlusal view. (d) Intraoral assessment of the coupled trays confirming stability and accuracy. (e) Intaglio view of the finalized SMART tray impression. (f) Occlusal perspective of the SMART tray impression. (g) Finished SMART tray impression before cast pouring. (h) SMART tray impression – Evaluation in the patient’s mouth. (i) Beading and boxing of the impression using the conventional plaster–pumice method reinforced with an OPG sheet.

The technique involved making impressions using a self-retaining custom tray. Trays 1 and 2 were first placed intraorally to verify proper seating and extension. The assembled tray was then positioned intraorally (Figure 3a) to establish a functional border seal. Sectional border molding was performed using a low-fusing impression compound (Pinnacle Tracing Stick, DPI, Mumbai, India) (Figure 3b–g). The patient was instructed to perform functional movements to shape labial and buccal borders. During border molding, the trays were stabilized using finger supports (rests) incorporated into the SMART tray handle design in Region-1. After completing border molding, both trays were detached to obtain the final impression. The placement and orientation of Trays 1 and 2 were rehearsed multiple times to ensure accurate recording of the knife-edge ridge area at rest without interference from the operator’s finger pressure. The impression of Region-1 was first made using zinc oxide eugenol wash impression material (DPI Impression Paste, Dental Products of India, Mumbai, India) (Figure 3h–4d), with stabilization provided by the handle intraorally. The tray was then removed, excess material was trimmed using sharp scissors, and the tray was reseated in the mouth.

Multiple holes were drilled in Tray-2 to enhance mechanical retention of the elastomeric impression material. Tray adhesive (Caulk Tray Adhesive, Dentsply India Private Limited, Mumbai, India) was applied, and light-body addition silicone (Aquasil Ultra LV, Dentsply India Private Limited, Mumbai, India) was gently syringed into Tray-2 (Figure 4e, f). The tray was oriented intraorally such that the opposing magnetic poles of Tray-1 and Tray-2 faced each other. Tray-2 was released when the magnetic pull was felt, and it was self-retained owing to the magnetic attraction, eliminating the need for additional finger pressure. Excess material escaped through the space between the two tray sections. The tray was held in place solely by a magnetic attraction. Once the impression material was fully polymerized, the entire tray assembly was removed from the patient’s mouth and the surface details were assessed (Figure 4g, h). To achieve well-defined master casts, the beading and boxing (Figure 4i) techniques were used for the final impressions. These methods were crucial for securely containing the impressions and ensuring the accurate and precise pouring of definitive casts. For this process, Type IV gypsum, specifically die stone (GypRock Dental Stone Class IV, Rajkot, Gujarat, India), was utilized to create definitive cast.

The denture base was constructed using a clear autopolymerizing acrylic resin (DPI RR Cold Cure, Dental Products of India, Mumbai, India). The base demonstrated a uniform fit with the knife-edge ridge area. To prevent tissue compression from uneven occlusal contacts, a static jaw-relation method using a bite registration material was employed. An occlusal scheme with minimal incisal guidance was selected, and acrylic teeth (Cosmo HXL, Dentsply India, Patparganj Industrial Area, Delhi, India) were modified to have minimal or nearly zero cusp degrees to reduce lateral forces. A wax try-in was performed, and the definitive complete dentures were processed using a heat-polymerizing denture base resin (Triplex Hot, Ivoclar Vivadent). Following processing, the dentures were finished, polished, and clinically evaluated for adaptation, retention, stability, esthetics, phonetics, and occlusion.

After the necessary occlusal adjustments were completed, the maxillary and mandibular complete dentures were inserted. The patient was given post-insertion instructions and was evaluated after three recall visits: 24 h, 1 week, and 1 month.

## Results

The SMART impression technique was successfully completed without any intraoperative complications. Magnetic self-retention of the split custom tray provided stable and reproducible positioning of the tray components during the definitive impression procedure, eliminating the need for manual finger pressure over the knife-edge residual ridge. The sectional impression procedure enabled accurate recording of both the stress-bearing tissues and the knife-edge ridge while maintaining the latter in a minimally displaced, mucostatic condition.

**Figure 5:**
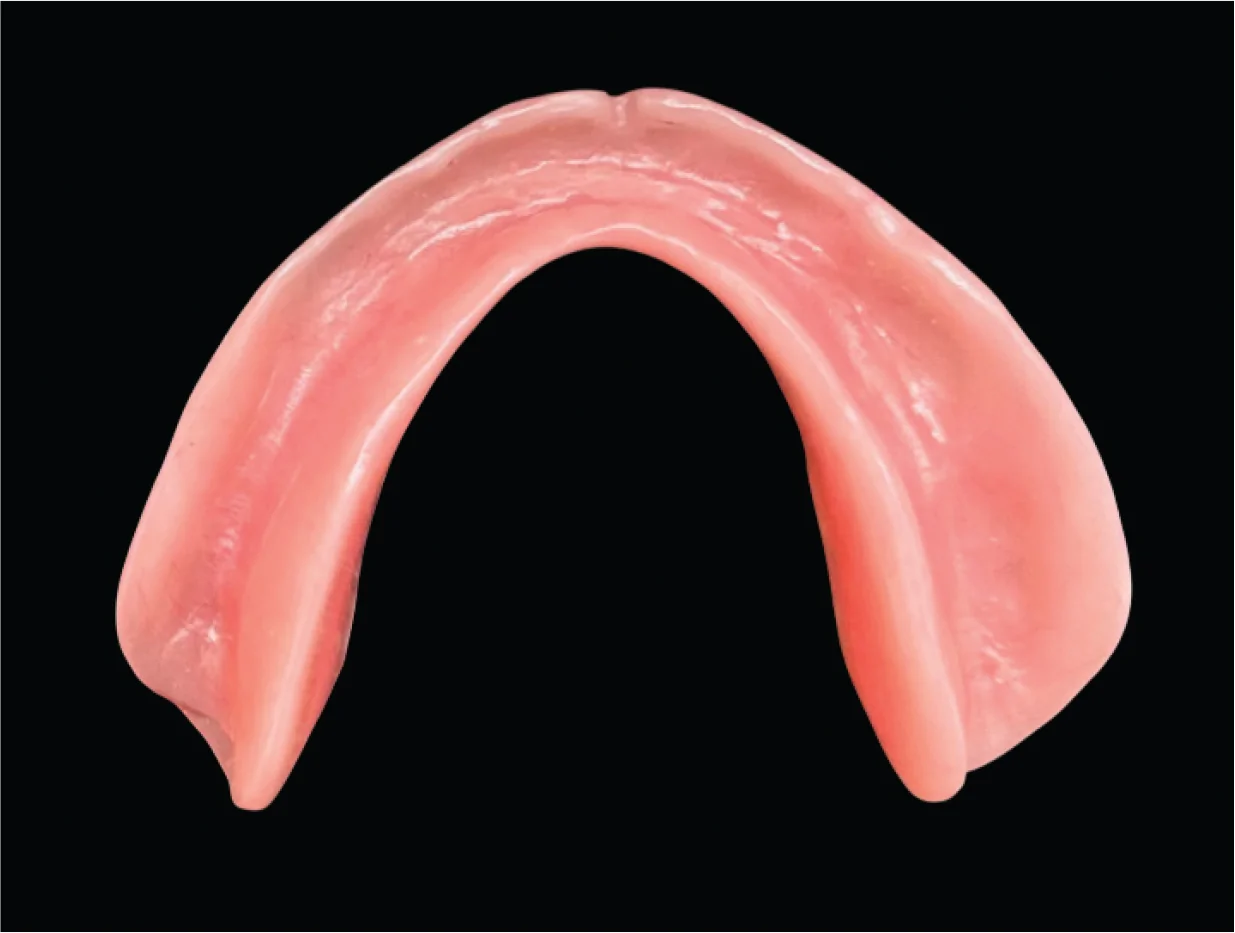
Intaglio (fitting) surface of the definitive mandibular complete denture demonstrating accurate adaptation to the knife-edge residual ridge anatomy reproduced using the SMART (Sikri’s Magnetic Attachment Retained Two-Tray) impression technique.

The definitive impression demonstrated accurate reproduction of the residual ridge anatomy with well-defined peripheral borders and satisfactory surface detail. Subsequent beading, boxing, and pouring produced a well-adapted master cast that accurately represented the clinical anatomy. The intaglio (fitting) surface of the definitive mandibular complete denture closely reproduced the contours of the residual ridge, indicating satisfactory tissue adaptation (Figure 5).

Following insertion, the maxillary and mandibular complete dentures exhibited satisfactory retention, stability, adaptation, esthetics, phonetics, and occlusal relationships. Minor occlusal adjustments were performed at insertion. Clinical evaluation during follow-up visits at 24 h, 1 week, and 1 month revealed no evidence of denture instability, excessive pressure over the knife-edge ridge, or clinically significant mucosal trauma requiring major prosthesis modification.

The patient reported improved comfort during denture wear, enhanced masticatory efficiency, and overall satisfaction with the treatment outcome. The chronological sequence of diagnosis, treatment, and follow-up is summarized in Table 1.

**Table 1:** Clinical timeline of the present case.

Clinical stage	Procedure performed	Clinical outcome
Visit 1	Comprehensive history taking, intraoral examination, and radiographic assessment	Complete edentulism with a pronounced mandibular anterior knife-edge residual ridge diagnosed. Patient deemed suitable for nonsurgical prosthodontic rehabilitation.
Visit 2	Primary impressions and fabrication of custom trays	Diagnostic casts obtained for treatment planning and fabrication of the SMART tray.
Laboratory Phase	Fabrication of the SMART (Sikri’s Magnetic Attachment Retained Two-Tray) split custom impression tray with incorporated magnetic attachments and retentive holes	Customized tray fabricated to facilitate passive tray stabilization and mucostatic impression making.
Visit 3	Definitive mandibular impression using the SMART tray technique with polyvinyl siloxane impression material	Accurate mucostatic impression obtained without manual finger pressure, demonstrating satisfactory reproduction of the knife-edge residual ridge anatomy.
Visit 4	Jaw relation records, face-bow transfer, and mounting on a semi-adjustable articulator	Maxillomandibular relationship accurately established and prosthetic rehabilitation planned.
Visit 5	Wax trial denture evaluation	Denture retention, stability, occlusion, aesthetics, and phonetics verified and accepted.
Visit 6	Processing, finishing, polishing, and insertion of definitive complete dentures	Complete dentures delivered with satisfactory retention, stability, support, and patient comfort.
Follow-up	Post-insertion evaluation and occlusal adjustments	Patient reported satisfactory comfort, improved masticatory efficiency, and absence of pain over the mandibular knife-edge residual ridge.

This case report has been prepared in accordance with the CARE (CAse REport) guidelines. A completed CARE Checklist has been submitted as supplementary material.

## Discussion

The present case demonstrated that the SMART impression technique enabled stable magnetic self-retention of the split custom tray, eliminated the need for manual finger pressure during impression making, and facilitated accurate mucostatic recording of a mandibular knife-edge residual ridge. The definitive denture exhibited satisfactory adaptation, retention, stability, and patient comfort. These findings suggest that magnetic self-retention may simplify impression making while minimizing tissue displacement in patients with challenging residual ridge anatomy.

The primary objective of complete denture rehabilitation is to restore oral function, comfort, aesthetics, and quality of life while preserving the remaining oral tissues. Achieving these objectives depends largely on the accuracy of the definitive impression, which directly influences denture support, stability, and retention. This becomes particularly challenging in patients with knife-edge residual ridges because the thin, sharp crest is covered by delicate mucosa that is highly susceptible to trauma during impression making and subsequent denture function [6, 14].

Successful prosthodontic management of knife-edge residual ridges extends beyond impression making and requires careful control of all biomechanical factors influencing denture stability. Appropriate orientation of the occlusal plane, selection of a suitable occlusal scheme, and establishment of balanced occlusion are essential to minimize destabilizing forces during function. In the present case, a face-bow transfer and mounting on a semi-adjustable articulator were performed to accurately reproduce the maxillomandibular relationship. Proper occlusal adjustment helps reduce lateral forces transmitted to the compromised residual ridge, thereby improving denture stability and patient comfort.

Several nonsurgical approaches have been proposed for managing knife-edge ridges, each aiming to reduce excessive pressure over the sharp crest while directing functional loads toward more favorable stress-bearing areas. As summarized in Table 2, the controlled-pressure impression technique described by Winkler [15] utilizes a custom tray with tracing compound to selectively relieve the fibrous crestal tissues before the final impression is made. This technique effectively redistributes occlusal forces toward primary stress-bearing regions, particularly the buccal shelf, but its application is generally limited to fibrous, non-resilient ridges. Similarly, Hyde [16] described the differential-pressure impression technique, in which perforations are created directly over the knife-edge ridge to reduce hydraulic pressure during impression making. This approach permits selective pressure distribution and helps preserve residual ridge height. However, the number, size, and distribution of tray perforations are largely operator dependent, potentially affecting the reproducibility of the impression procedure and the consistency of clinical outcomes.

**Table 2:** Prosthodontic impression techniques for the management of knife-edge residual ridges.

S. No.	Prosthodontic technique	Author(s)	Description (methodology)	Advantages	Limitations/issues
1	Controlled-pressure impression technique	Winkler	The technique uses a custom tray designed to control the pressure exerted on the denture-bearing tissues. A tracing compound (green stick) is used to identify and record the denture-bearing area. The green stick is subsequently removed from the region corresponding to the fibrous crestal tissues, and the perforated area is enlarged before making the definitive impression. This arrangement reduces pressure over the knife-edge crest while allowing greater loading of more favorable primary stress-bearing areas.	Reduces direct occlusal loading over the affected crestal region; directs functional forces toward more favorable supporting tissues, particularly the buccal shelf; relatively simple and clinically applicable.	Primarily indicated when the crestal tissues are fibrous and non-resilient; accurate identification and relief of the affected area are operator dependent.
2	Differential-pressure impression technique	T. P. Hyde	A custom tray is modified to provide differential pressure over the denture-bearing tissues. The area corresponding to the knife-edge ridge is delineated and relieved, and multiple small perforations are created directly over the sharp ridge. During impression making, these perforations allow escape of impression material and reduce pressure over the vulnerable crestal tissues while permitting greater pressure over more favorable supporting areas.	Minimizes excessive pressure over the sharp ridge; facilitates preservation of residual ridge height; permits selective distribution of impression pressure toward more favorable supporting tissues.	The number, size, and location of perforations are not standardized; modification is largely operator dependent and may vary according to clinical judgment and experience.
3	Selective relief/perforation technique for knife-edge ridge	Various authors	Localized relief is incorporated into the custom tray directly over the knife-edge crest. Depending on the clinical situation, the relief may be produced by selective reduction of the tray, removal of spacer material, or creation of one or more perforations over the sharp ridge. The definitive impression is then made with controlled pressure over the remaining denture-bearing tissues.	Simple, inexpensive, and readily incorporated into conventional custom-tray procedures; decreases localized pressure over the sharp ridge; allows preservation of the available supporting anatomy.	The amount and location of relief are not universally standardized; inadequate relief may result in tissue compression, whereas excessive relief may compromise impression control and support.
4	Mucostatic impression approach for knife-edge ridge	Boucher; subsequent clinical applications	The impression is made with the objective of recording the knife-edge residual ridge in a minimally displaced state. A custom tray with appropriate relief is used, and a low-viscosity impression material is manipulated with minimal seating pressure to reduce displacement of the thin mucosa overlying the sharp ridge.	Minimizes tissue displacement; potentially reduces discomfort associated with compression of the thin mucosa over the sharp ridge; preserves the recorded morphology of the delicate residual ridge.	Complete elimination of tissue distortion is not possible because tray seating, material flow, and clinical manipulation inevitably generate some pressure; careful tray adaptation and controlled seating are required.
5	ASKER (Arpit Sikri Knife-Edge Ridge) impression technique	Arpit Sikri and Jyotsana Sikri	A single-step impression technique using polyvinyl siloxane is performed with a custom tray fabricated from light-cured acrylic resin. A customized commercially available prefabricated metal mesh is incorporated into the tray to facilitate controlled support and management of impression-material thickness over the knife-edge ridge. The technique is designed to minimize pressure over the sharp residual ridge while facilitating accurate reproduction of the denture-bearing tissues.	Simple and efficient; single-step impression procedure; minimizes procedural complexity and potential errors; facilitates complete denture fabrication without additional chairside visits; uses conventional clinical procedures and does not require specialized equipment or auxiliary personnel; economically feasible.	Requires intricate customization and accurate positioning of the metal mesh; precise control of impression-material thickness is necessary; removal of a conventional wax spacer may present practical difficulties; technique is operator dependent.
6	SMART (Sikri’s Magnetic Attachment Retained Two-Tray) impression technique	Arpit Sikri	The SMART technique incorporates magnetic self-retention into a split, two-part custom tray. Small magnetic attachments are incorporated into the tray components to facilitate passive attraction and reproducible reorientation during impression making. Following appropriate tray preparation and border molding, the impression material is loaded into the tray and the two components are approximated through magnetic attraction. This design minimizes the need for manual stabilization or finger pressure during tray seating and facilitates recording of the knife-edge residual ridge in a minimally displaced, mucostatic condition.	Provides passive and reproducible tray stabilization through magnetic attraction; minimizes operator-applied finger pressure and tissue displacement; facilitates controlled recording of the sharp residual ridge; combines pressure control with improved tray stabilization; simplifies clinical handling; can be incorporated into routine complete denture fabrication without specialized equipment or additional appointments.	Accurate positioning and orientation of the magnetic attachments are essential; uniform spacer adaptation and controlled impression-material thickness are required; improper alignment may produce localized tissue compression or compromise impression accuracy; currently supported by a single clinical case and requires validation through larger clinical studies and long-term follow-up.

A related approach involves selective relief or perforation of the custom tray over the knife-edge region to minimize localized pressure during impression making [17, 18]. Boucher described the use of relief over a mandibular knife-edge ridge to prevent excessive contact and displacement of the vulnerable crestal tissues while maintaining adequate contact with the remaining denture-bearing areas [17]. In clinical application, this principle may be achieved through selective reduction of the tray or spacer, or by incorporating perforations over the corresponding ridge area to allow escape of excess impression material and thereby reduce localized pressure. Although relatively simple and inexpensive, the effectiveness of this approach depends on accurate identification of the knife-edge region and appropriate determination of the extent of relief. Furthermore, the dimensions and distribution of relief or perforations are not uniformly standardized, making the technique susceptible to operator-dependent variations.

The mucostatic impression concept provides another rationale for managing a knife-edge residual ridge by attempting to record the denture-bearing tissues with minimal displacement. Addison introduced the mucostatic impression concept, emphasizing the recording of oral tissues in a minimally displaced state [19]. Boucher subsequently incorporated the principle of selective relief into complete denture impression procedures and specifically advocated relief when a mandibular knife-edge ridge was present [20, 21]. In the context of a knife-edge ridge, a mucostatic approach may be advantageous because the thin mucosa overlying the sharp residual ridge is particularly susceptible to compression and discomfort. The use of an appropriately relieved custom tray, a low-viscosity impression material, and minimal seating pressure may therefore reduce unnecessary tissue displacement and facilitate accurate reproduction of the delicate ridge anatomy. However, complete elimination of tissue displacement cannot be achieved because some degree of pressure inevitably results from tray seating, impression-material flow, border molding, and clinical manipulation. Moreover, mucostatic recording primarily addresses tissue displacement and does not independently resolve the issue of maintaining consistent and reproducible tray positioning during impression making.

More recently, the ASKER (Arpit Sikri Knife-Edge Ridge) impression technique was introduced as a simplified single-step approach utilizing a customized metal mesh incorporated into a light-cured acrylic custom tray together with polyvinyl siloxane impression material [22]. The technique was designed to simplify the clinical procedure, reduce chairside time, and eliminate the need for additional appointments while maintaining satisfactory impression accuracy. Nevertheless, the customization of the metal mesh, precise control of impression material thickness, and removal of the wax spacer remain technique-sensitive steps that require clinical experience.

The SMART impression technique presented in this report differs from the previously described methods by incorporating magnetic self-retention within a split two-part custom tray. Unlike conventional techniques that require manual stabilization throughout impression making, magnetic attraction between the tray components provides passive and reproducible positioning without the application of finger pressure. This design minimizes operator-induced tissue displacement and facilitates recording of the residual ridge in a minimally displaced, mucostatic condition. Consequently, the technique combines the advantages of pressure control with improved tray stabilization and simplified clinical handling.

The biomechanical rationale of the SMART impression technique is based on the principle of minimizing functional stress over the sharp residual ridge while directing occlusal loads toward more favorable denture-bearing tissues, particularly the buccal shelf. Excessive compression of the thin mucosa covering a knife-edge ridge may accelerate discomfort, tissue injury, and further RRR. Recording the supporting tissues with minimal displacement therefore contributes to improved adaptation of the denture base and may enhance patient comfort during function [23].

Although the concept of mucostatic impression making has been debated for many years, its fundamental principle remains applicable in situations where tissue displacement should be minimized. Complete elimination of tissue distortion is difficult because impression trays, impression materials, and clinical manipulation inevitably produce some degree of pressure. Nevertheless, minimizing operator-applied forces during impression making may reduce unnecessary tissue displacement and improve the accuracy of recording delicate residual ridge anatomy. To the best of our knowledge, no previously published impression technique for knife-edge residual ridges has utilized magnetic self-retention to eliminate manual tray stabilization during impression making.

Selection of an appropriate impression material is equally important for obtaining an accurate definitive impression. Spacer thickness should correspond to the rheological characteristics of the selected impression material, with approximately 1.3 mm recommended for light-body elastomers, 3 mm for medium-body elastomers and irreversible hydrocolloids, and 0.5 mm for zinc oxide-eugenol impression paste [24]. In the present technique, polyvinyl siloxane was selected because of its excellent dimensional stability, elastic recovery, tear resistance, handling characteristics, and ability to accurately reproduce fine surface details while minimizing distortion during removal [25].

An additional advantage of the SMART tray impression technique is its clinical simplicity. The procedure can be incorporated into routine complete denture fabrication without increasing the number of clinical appointments or requiring specialized equipment beyond the incorporation of small magnetic attachments into the custom tray. Consequently, the technique may be readily adopted in general prosthodontic practice while remaining economically feasible.

Despite these advantages, certain limitations should be recognized. Accurate positioning of the magnetic attachments is essential to ensure precise reorientation of the split tray components. Uniform wax spacer adaptation and controlled impression material thickness are also necessary to maintain the intended mucostatic effect. Inaccuracies in these steps may result in localized tissue compression and compromise impression accuracy. Furthermore, the present report describes a single clinical case; therefore, the reproducibility and long-term clinical performance of the SMART technique require validation through prospective clinical studies involving larger patient populations.

Overall, comparison with previously published impression techniques demonstrates that the SMART tray impression technique shares the common objective of minimizing stress on the knife-edge residual ridge while introducing magnetic self-retention as a novel method of tray stabilization. This design simplifies impression making, reduces operator-dependent pressure during tray seating, and may improve the accuracy of mucostatic impressions in patients presenting with challenging mandibular knife-edge residual ridges.

Various impression techniques for the effective prosthodontic management of knife-edge ridges are presented in Table 2.

## Conclusion

The SMART impression technique provided a simple and practical method for recording a mandibular knife-edge residual ridge in the present case. The magnetically retained split-tray design enabled stable tray positioning without manual finger pressure, facilitating a mucostatic impression and accurate recording of the residual ridge anatomy. This technique may serve as a useful nonsurgical alternative for selected patients with knife-edge residual ridges; however, further clinical studies are required to evaluate its reproducibility and long-term clinical effectiveness.

## Data Availability

All data generated or analyzed during this study are included in this published article. Additional information is available from the corresponding author upon reasonable request.
